# Sodium Iodate-Induced Ferroptosis in Photoreceptor-Derived 661W Cells Through the Depletion of GSH

**DOI:** 10.3390/ijms26052334

**Published:** 2025-03-05

**Authors:** Chao Chen, Han Wang, Jiuyu Yang, Bi Zhao, Yutian Lei, Hanqiao Li, Kunhuan Yang, Benying Liu, Yong Diao

**Affiliations:** 1 Institute of Genomics, School of Medicine, Huaqiao University, 668 Jimei Road, Xiamen 361021, China; 22013071025@stu.hqu.edu.cn (H.W.); 22013071032@stu.hqu.edu.cn (J.Y.); 23013071010@stu.hqu.edu.cn (Y.L.); diaoyong@hqu.edu.cn (Y.D.); 2 Yunnan Key Laboratory of Tea Science, Tea Research Institute, Yunnan Academy of Agricultural Science, Kunming 650201, China; liubenying5812@foxmail.com; 3 School of Medicine, Xiamen University, Xiamen 361000, China; 24520231154823@stu.xmu.edu.cn (H.L.); kunhuan2024@foxmail.com (K.Y.)

**Keywords:** sodium iodate, photoreceptor, ferroptosis, GSH

## Abstract

Oxidative stress-induced photoreceptor cell death is closely associated with the etiology of age-related macular degeneration (AMD), and sodium iodate (SI) has been widely used as an oxidant stimulus in AMD models to induce retinal pigment epithelium (RPE) and photoreceptor cell death. However, the mechanism underlying SI-induced photoreceptor cell death remains controversial and unclear. In this study, we elucidate that ferroptosis is a critical form of cell death induced by SI in photoreceptor-derived 661W cells. SI disrupts system Xc^−^, leading to glutathione (GSH) depletion and triggering lipid peroxidation, thereby promoting ferroptosis in photoreceptor-derived 661W cells. Additionally, SI enhances intracellular Fe^2+^ levels, which further facilitates reactive oxygen species (ROS) accumulation, making the 661W cells more susceptible to ferroptosis. Exogenous GSH, as well as specific inhibitors of ferroptosis such as Fer-1 and antioxidants like NAC, significantly attenuate SI-induced ferroptosis in photoreceptor-derived 661W cells. These findings provide new insights into the mechanisms of ferroptosis as a key pathway in SI-induced photoreceptor-derived 661W cell death.

## 1. Introduction

Age-related macular degeneration (AMD) is a leading cause of vision impairment in the elderly [1]. AMD is classified into dry and wet types [2]. Wet AMD is defined by choroidal neovascularization [3], leading to significant vision loss, whereas dry AMD, accounting for over 80% of cases [4], is characterized by macular geographic atrophy without neovascularization [5]. A European study showed that antivascular endothelial growth factor (VEGF) therapies decrease AMD prevalence in patients with choroidal neovascularization (CNV) [6]. However, treatment options for dry AMD are limited, with no alternatives that achieve the significant efficacy of anti-angiogenic therapies used for wet AMD [7,8]. Therefore, global projections estimate that AMD cases will rise to 300 million by 2040 [9], emphasizing its persistent threat to global visual health [10].

The pathogenesis of AMD involves multifactorial contributors, including age [1,11], genetics [12,13], inflammation [4,14], oxidative stress [15,16], and environmental factors like diet and smoking [17]. Among these, the death of photoreceptor cells and retinal pigment epithelial (RPE) cells caused by these factors is closely associated with AMD progression [18,19]. Particularly, oxidative stress-induced damage to RPE and photoreceptor cells is a key driver of AMD development [20,21]. It has been suggested that oxidative stress induces inflammation [22], necroptosis [16,23], apoptosis [24,25], and pyroptosis [26] in retinal cells, worsening retinal degeneration. In addition, excessive reactive oxygen species (ROS) accumulation triggers ferroptosis, a form of Fe^2+^-dependent cell death, by driving lipid peroxidation [27,28,29]. Intriguingly, ferroptosis has been increasingly associated with AMD pathology, and its inhibition shows promise as a therapeutic strategy [30,31,32]. Sodium iodate (SI), an oxidative agent, is commonly used in cellular and animal AMD models to study retinal degeneration and assess potential treatments [33,34,35,36]. Mechanistic studies reveal that SI upregulates iron levels in RPE cells, depletes intracellular glutathione (GSH), and increases lipid peroxidation, collectively driving ferroptosis [33,37,38,39]. Despite its widespread use to model retinal degeneration, the effects of SI on photoreceptor cell death are poorly understood. Furthermore, whether ferroptosis contributes to SI-induced photoreceptor cell death also remains unclear.

In this study, we demonstrated that SI induces ferroptosis in photoreceptor-derived 661W cells (661W cells) primarily through the depletion of GSH. Moreover, the use of the ferroptosis inhibitor Ferrostatin-1(Fer-1), as well as the antioxidants *N*-Acetyl-*L*-cysteine (NAC) and GSH, significantly alleviated SI-induced ferroptosis in photoreceptor-derived 661W cells.

## 2. Results

### 2.1. SI Induces Ferroptosis as One of the Cell Death Pathways in Photoreceptor-Derived 661W Cells

**Figure 1:**
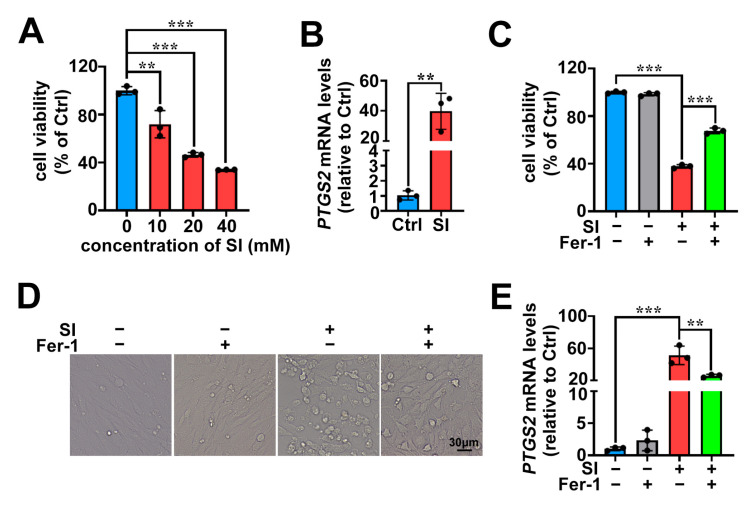
The ferroptosis inhibitor Fer-1 attenuates SI-induced cell death in photoreceptor-derived 661W cells. (**A**) Cell viability was measured using the MTS assay in 661W cells after treatment with SI at concentrations of 10, 20, and 40 mM for 18 h (hours). (**B**) The mRNA levels of *PTGS2* in 661W cells at a concentration of 20 mM SI for 18 h. (**C**) Cell death was assessed using an MTS assay in 661W cells pre-treated with 10 μM Fer-1 and then treated with 20 mM SI for 18 h. (**D**) Cell morphology was observed in images after pre-treatment with 10 μM Fer-1 and treatment with 20 mM SI for 18 h. Scale bars = 30 μm. (**E**) The *PTGS2* mRNA levels in 661W cells pre-treated with 10 μM Fer-1 then treated with 20 mM SI for 18 h. Statistical analyses were assessed using Student’s *t*-test in (**B**), and the statistical analyses for (**A**,**C**,**E**) were conducted using one-way ANOVA with Tukey’s multiple comparison test. ** *p* < 0.01; *** *p* < 0.001.

The results of MTS assays, shown in Figure 1A, demonstrated that SI induced the death of 661W cells in concentration-dependent manner. Treating 661W cells with SI for 18 h at a concentration of 20 mM resulted in a significant reduction in cell viability of almost 53.5%. On the basis of the MTS data, SI at the concentration of 20 mM was exposed to 661W cells for 18 h in subsequent experiments. A previous report has disclosed that SI induces apoptosis in 661W cells [40]. However, to further certify that apoptosis was not the only cause of photoreceptor cell death induced by SI, we employed qRT-PCR to examine the expression of the ferroptosis-related marker gene *PTGS2*. The results showed that treatment with 20 mM SI significantly upregulated *PTGS2* mRNA levels in 661W cells (Figure 1B). Fer-1 is recognized as a selective ferroptosis inhibitor [41]. In this study, we observed that treatment with Fer-1 at concentrations of 10 μM partially protected 661W cells from SI-induced cell death (Figure 1C). As shown by microscope, Fer-1 treatments also obviously mitigated cell cytoplasmic rupture and morphological changes by SI (Figure 1D). Additionally, the expression levels of ferroptosis-related marker *PTGS2* mRNA were significantly reduced following Fer-1 treatment (Figure 1E). These findings suggest that ferroptosis partially contributes to SI-induced photoreceptor-derived 661W cell death.

### 2.2. SI Treatment Leads to Lipid Peroxidation in Photoreceptor-Derived 661W Cells

**Figure 2:**
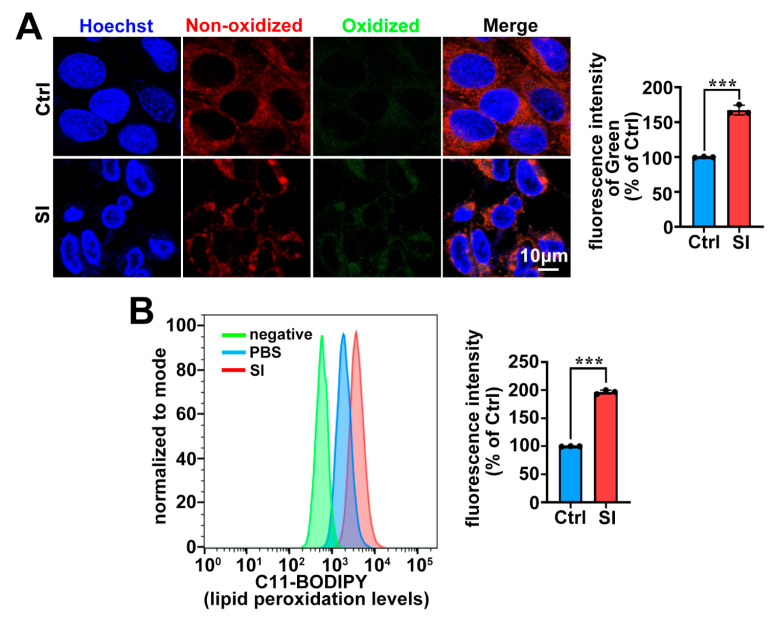
SI results in lipid peroxidation in photoreceptor-derived 661W cells. (**A**) Lipid peroxidation analyzed using C11-BODIPY (Green) and visualized by fluorescence microscopy in 661W cells after 18 h of 20 mM SI treatment. Scale bars = 10 μm. Quantification of green fluorescence using ImageJ software (version 2.9.0). (**B**) Lipid peroxidation detected using C11-BODIPY staining and quantified by flow cytometry in 661W cells treated with 20 mM SI for 18 h. Statistical analysis of (**A**,**B**) performed using Student’s *t*-test. *** *p* < 0.001.

Lipid peroxidation is a key hallmark of ferroptosis [29,42]. In this work, we employed C11-BODIPY staining combined with confocal microscopy and flow cytometry to quantify lipid peroxidation in 661W cells. As expected, lipid peroxidation was significantly upregulated in 661W cells treated with 20 mM SI (Figure 2). These results suggest that SI exacerbates lipid peroxidation, thereby inducing ferroptosis in photoreceptor-derived 661W cells.

### 2.3. SI Elevates Intracellular Fe^2+^ and ROS Levels in Photoreceptor-Derived 661W Cells

**Figure 3:**
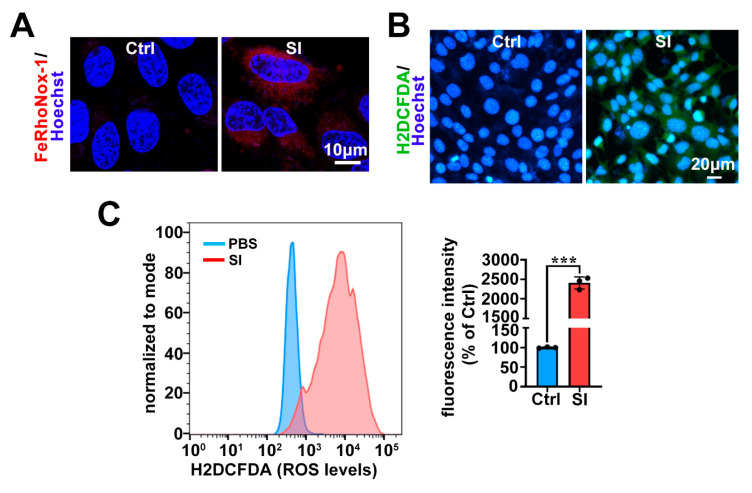
Elevated intracellular Fe^2+^ and ROS levels in SI-treated photoreceptor-derived 661W cells. (**A**) Intracellular Fe^2+^ levels in 661W cells measured using fluorescent probe FeRhoNox-1 (Red) after 18 h of treatment with 20 mM SI. Scale bars = 10 μm. (**B**) Intracellular ROS levels in 661W cells assessed using fluorescent probe H2DCFDA (Green) following 18 h of 20 mM SI treatment. Scale bars = 20 μm. (**C**) Intracellular ROS levels quantified using H2DCFDA staining and analyzed via flow cytometry in 661W cells treated with 20 mM SI for 18 h. Statistical analysis for (**C**) conducted using Student’s *t*-test. *** *p* < 0.001.

Previous studies have shown that excess intracellular Fe^2+^ can trigger the accumulation of ROS through the Fenton reaction, thereby facilitating ferroptosis [29,43]. Using the FeRhoNox-1 probe, we observed an increase in intracellular Fe^2+^ levels in 661W cells following SI overload (Figure 3A). Moreover, we employed the H2DCFDA probe together with confocal microscopy and flow cytometry to quantify intracellular ROS accumulation, revealing a significant increase in ROS levels. (Figure 3B,C). Based on these results, we conclude that SI promotes ferroptosis in photoreceptor-derived 661W cells by elevating intracellular Fe^2+^ and exacerbating ROS accumulation.

### 2.4. The Antioxidant NAC Protects Photoreceptor-Derived 661W Cells from SI-Induced Ferroptosis

**Figure 4:**
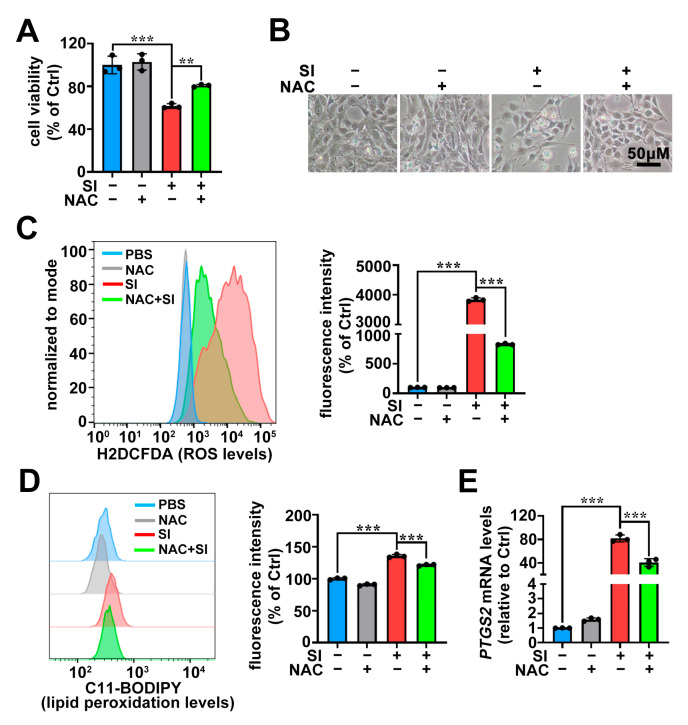
The antioxidant NAC alleviates SI-induced ferroptosis in photoreceptor-derived 661W cells. (**A**) Cell viability was assessed by MTS; 661W cells were pre-treated with 2 mM NAC and then treated with 20 mM SI for 18 h. (**B**) Cell morphology changes were observed in images captured from 661W cells pre-treated with 2 mM NAC and then treated with 20 mM SI for 18 h. Scale bars = 50 μm. (**C**) Intracellular ROS levels were analyzed using H2DCFDA (Green) staining, followed by flow cytometry in 661W cells pre-treated with 2 mM NAC and then treated with 20 mM SI for 18 h. (**D**) Lipid peroxidation levels were assessed using C11-BODIPY in 661W cells pre-treated with 2 mM NAC and then treated with SI for 18 h. Flow cytometry was used for analysis, and the relative intensity was quantified using FlowJo software (version 10). (**E**) qRT-PCR was used to analyze the mRNA levels of *PTGS2* in 661W cells pre-treated with 2 mM NAC and then treated with SI for 18 h. One-way ANOVA with Tukey’s multiple comparison test was performed for the statistical analyses of (**A**,**C**–**E**). ** *p* < 0.01; *** *p* < 0.001.

To further confirm that SI-induced ROS accumulation facilitates ferroptosis in 661W cells, we treated the cells with 2 mM of the antioxidant NAC. As shown in Figure 4A, NAC treatment significantly rescued SI-induced 661W cell death, with remarkable improvements in cellular morphology and reduced damage (Figure 4B). Flow cytometry using the H2DCFDA probe demonstrated a marked decrease in intracellular ROS levels following NAC treatment (Figure 4C). C11-BODIPY staining revealed a significant reduction in lipid peroxidation in SI-treated cells upon NAC intervention (Figure 4D). Moreover, the expression of the *PTGS2* mRNA was significantly downregulated (Figure 4E). These findings further support the conclusion that SI-induced intracellular ROS elevation drives ferroptosis in photoreceptor-derived 661W cells.

### 2.5. System Xc^−^/GSH/GPX4 Axis in SI-Induced Photoreceptor-Derived 661W Cell Ferroptosis

**Figure 5:**
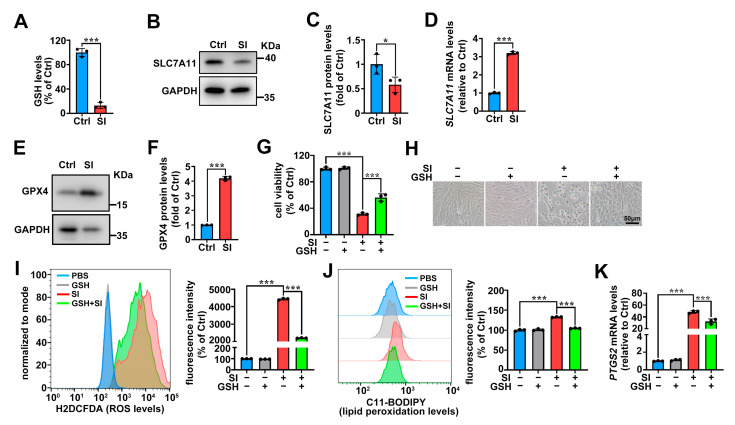
SI causes GSH depletion in photoreceptor-derived 661W cells. (**A**) Intracellular GSH levels were measured using the GSH/GSSG assay kit in 661W cells treated with 20 mM SI for 18 h. (**B**,**C**), Western blot analysis was performed to assess the levels of SLC7A11 protein in 661W cells treated with 20 mM SI for 18 h. GAPDH served as the loading control, and the relative intensity was quantified using ImageJ software. (**D**) *SLC7A11* mRNA expression levels were assessed by qRT-PCR in 661W cells after 18 h of 20 mM SI treatment. (**E**,**F**) Western blot analyses were performed to assess the levels of GPX4 proteins in 661W cells treated with 20 mM SI for 18 h. GAPDH served as the loading control, and the relative intensity was quantified using ImageJ software. (**G**) Cell viability was assessed using the MTS assay kit; 661W cells were pre-treated with 50 μM GSH and then treated with 20 mM SI for 18 h. (**H**) Images were captured after 50 μM GSH pre-treatment to observe changes in cell morphology. The scale bars indicate 50 μm. (**I**) After pre-treatment with 50 μM GSH, the 661W cells were treated with 20 mM SI for 18 h, and the intracellular ROS levels were analyzed by flow cytometry, the relative intensity was quantified using FlowJo software. (**J**) The cells were pre-treated with 50 μM GSH, then treated with 20 mM SI for 18 h, stained with C11-BODIPY, and analyzed for lipid peroxidation by flow cytometry. The relative intensity was quantified using FlowJo software. (**K**) qPCR analysis showed alterations in *PTGS2* gene expression; 661W cells were pre-treated with 50 μM GSH and then treated with 20 mM SI for 18 h. The statistical analyses were assessed using Student’s *t*-test in (**A**,**C**,**D**,**F**). * *p* < 0.05; *** *p* < 0.001. One-way ANOVA with Tukey’s multiple comparison test was performed for the statistical analyses of (**G**,**I**–**K**). *** *p* < 0.001.

The depletion of GSH leads to GPX4 inactivation, triggering lipid peroxidation and facilitating ferroptosis [28,29,43]. The cystine/glutamine antiporter (system X_C_^−^) is responsible for intracellular GSH synthesis [28]. Herein, we found that SI significantly depletes GSH levels in 661W cells (Figure 5A). Notably, SI-treated 661W cells showed reduced SLC7A11 protein expression (Figure 5B,C), a key component of system Xc^−^. However, *SLC7A11* mRNA expression was upregulated in SI-treated 661W cells (Figure 5D). Western blot analysis revealed a significant upregulation of GPX4 protein expression (Figure 5E,F). Additionally, the MTS assay results showed that supplementation with 50 μM of exogenous GSH alleviated the 661W cell death induced by 20 mM SI (Figure 5G,H). More importantly, GSH treatment significantly reduced intracellular ROS production and lipid peroxidation levels (Figure 5I,J), and also downregulated *PTGS2* mRNA expression levels in SI-loaded 661W cells (Figure 5K). Taken together, these findings provide evidence that SI induces ferroptosis in photoreceptor-derived 661W cells by impairing GSH synthesis.

## 3. Discussion

Normal photoreceptors are the foundation of the visual cycle and vision [44]. Mammalian photoreceptors consist of cones and rods, with the macula, the region of sharpest vision, being rich in cone cells [45,46]. In patients with AMD, macular degeneration is a key pathological feature [47]. Consequently, photoreceptor atrophy and death are closely associated with AMD [20,21]. SI, a chemical inducer, is widely used to establish experimental cell and animal models for studying retinal cell damage and AMD pathology [48]. Currently, SI is predominantly employed to provoke damage in RPE cells, which are adjacent to photoreceptors [49]. Recently, it was reported that SI triggers ferroptosis in human retinal pigment epithelium ARPE-19 cells, indicating that ferroptosis plays a significant role in SI-induced RPE cell death [33]. Although SI has been shown to cause apoptosis in photoreceptor-derived 661W cells [40,50], it remains unclear whether SI can also prompt ferroptosis in photoreceptor-derived 661W cells. In this work, we demonstrated that the widely used ferroptosis inhibitor Fer-1 significantly suppressed SI-driven photoreceptor-derived 661W cell death (Figure 1C,D), suggesting that ferroptosis is involved in the mechanism of SI-provoked photoreceptor-derived 661W cell damage.

Ferroptosis is a non-apoptotic form of cell death characterized by lipid peroxidation driven by ROS accumulation in an iron-dependent manner [29]. Our recent research revealed that all-*trans*-retinal (atRAL) accumulation, caused by disruptions in the visual cycle, can initiate ferroptosis in photoreceptor cells, highlighting the relevance of ferroptosis in AMD pathology [30]. In this study, we also observed substantial Fe^2+^ accumulation in SI-treated photoreceptor-derived 661W cells (Figure 3A). Crucially, significant ROS production and lipid peroxidation were detected (Figure 2 and Figure 3B,C). It is well known that oxidative stress induced by ROS can promote the release of Fe^2+^ from intracellular stores, such as ferritin [51]. Therefore, the ROS accumulation in 661W cells induced by SI further promotes the accumulation of intracellular Fe^2+^, which in turn also contributes to ROS accumulation, thereby promoting SI-induced ferroptosis in photoreceptor-derived 661W cells. Furthermore, the antioxidant NAC mitigated lipid peroxidation and reduced ROS production, thereby alleviating SI-induced photoreceptor-derived 661W cell damage and death (Figure 4A–D). It is well-established that during ferroptosis, intracellular expression of the *PTGS2* gene is significantly upregulated [52,53]. We also observed notable increases in *PTGS2* mRNA levels in SI-exposed 661W cells (Figure 1B). Both Fer-1 and NAC significantly downregulated *PTGS2* mRNA expression (Figure 1E and Figure 4E); these results provide further evidence that SI induced ferroptosis in photoreceptor-derived 661W cells.

**Figure 6:**
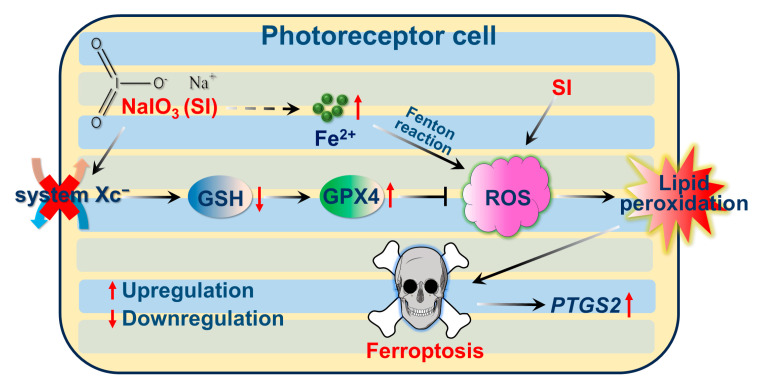
Schematic representation of mechanisms of SI-induced ferroptosis in photoreceptor-derived 661W cells, involving increased Fe^2+^ accumulation and inhibition of system X_C_^−^, leading to GSH depletion, ROS exacerbation, and lipid peroxidation.

Erastin and RSL3 are classical chemical triggers of ferroptosis [43]. Erastin disrupts the system Xc^−^ cystine–glutamate antiporter, inhibiting SLC7A11 and subsequently blocking GSH synthesis [29,54]; this leads to GPX4 inactivation, resulting in lipid peroxidation and ferroptosis [43]. In contrast, RSL3 directly inhibits GPX4, thereby promoting lipid peroxidation and ferroptosis [55]. In our study, SLC7A11 protein expression was indeed reduced in SI-treated photoreceptor cells (Figure 5B,C), and SI exposure caused GSH depletion in photoreceptor-derived 661W cells (Figure 5A). However, supplementation with exogenous GSH significantly reduced SI-induced ROS levels, lipid peroxidation, and photoreceptor-derived 661W cell death (Figure 5G–J). Additionally, GSH treatment downregulated *PTGS2* mRNA expression (Figure 5K). We also observed the upregulation of *SLC7A11* mRNA and GPX4 protein levels in SI-treated photoreceptor cells (Figure 5D–F), consistent with findings in studies of PM2.5-induced ferroptosis in human endothelial cells [56]. This upregulation may result from a compensatory negative feedback mechanism, wherein SI-induced depletion of GSH by the inhibition of system X_C_^−^ triggers increased *SLC7A11* mRNA expression, and GPX4 protein expression is enhanced to counteract excessive ROS and lipid peroxidation. Based on these results, we propose a molecular mechanism for SI-induced ferroptosis in photoreceptor-derived 661W cells, illustrated in Figure 6.

In summary, we revealed that SI induces ferroptosis in photoreceptor-derived 661W cells through the system Xc^−^/GSH/GPX4 axis. Interestingly, we found that a recent study also reported SI-induced ferroptosis in photoreceptor cells, both in vivo and in vitro [48], which aligns with our findings in vitro. Our research extends this work by employing a different experimental approach, while providing further validation and insights into the specific mechanisms underlying SI-induced ferroptosis in photoreceptor-derived 661W cells. Notably, treatment with Fer-1/GSH/NAC did not fully protect the 661W cell line from SI-induced cell death (Figure 1C,D, Figure 4A,B and Figure 5G,H). This suggests that, in addition to inducing ferroptosis, SI may involve other potential cell death mechanisms in photoreceptor-derived 661W cells. For instance, studies have reported that SI can also trigger apoptosis in 661W cells [50]. Therefore, future research should further explore the various cell death pathways induced by SI and their potential interactions in 661W cells. Additionally, in vivo studies are needed to further elucidate the molecular mechanisms underlying SI-mediated ferroptosis in photoreceptor cells.

## 4. Materials and Methods

### 4.1. Materials

SI (catalog no. A9165), Fer-1 (catalog no. SML0583), NAC (catalog no. A7250), GSH (catalog no. PHR1359), Hoechst 33342, and C11 BODIPY^581/591^ were obtained from Sigma-Aldrich (Saint Louis, MO, USA). FeRhoNox-1 was obtained from Goryo Chemical (Sapporo, Japan).

### 4.2. Cell Culture, Viability Assay, and Morphology Assessment

Photoreceptor-derived 661W cell lines, which were murine in origin, were procured from Shanghai Zishi Biotechnology (Shanghai, China) and maintained under standard culture conditions as described previously [57]. Cell viability was evaluated by the CellTiter 96^®^ AQueous One Solution (MTS) proliferation assay (Promega; Madison, WI, USA). The specific procedural method was as previously described [57]. Briefly, 661W cells were plated in 96-well plates and treated with the respective drugs. Following treatment, 20 µL of CellTiter 96 AQueous One Solution Reagent (Promega; Madison, WI, USA) was added to each well. After a 30 min incubation, absorbance was measured at 490 nm using a Varioskan™ LUX Multimode Microplate Reader (Thermo Fisher Scientific, Vantaa, Finland). Cell morphology was captured using a Leica microscope (Leica; Wetzlar, Germany).

### 4.3. Measurement of GSH Levels

The evaluation of GSH levels was conducted utilizing a GSH and GSSG assay kit (Beyotime; Shanghai, China) in accordance with the manufacturer’s instructions. The experimental procedure was as previously reported [30]. Briefly, 661W cells were seeded in 6-well plates and treated with 20 mM SI or vehicle (PBS) for 18 h. After two PBS washes, cells were scraped, centrifuged, and resuspended in protein removal reagent M. Following two freeze–thaw cycles, samples were centrifuged at 10,000× *g* for 10 min. The supernatant was transferred to a 96-well plate, and 150 µL of glutathione assay reagent and 50 µL of NADPH (0.5 mg/mL) were added. Absorbance at 412 nm was measured using a Varioskan™ LUX Multimode Microplate Reader (Thermo Fisher Scientific, Vantaa, Finland).

### 4.4. Quantitative Reverse Transcription Polymerase Chain Reaction (qRT-PCR)

Total RNA was extracted from photoreceptor cells using TRNzol Universal Reagent (TIANGEN, Beijing, China). Subsequently, RNA was reverse-transcribed into cDNA following the manufacturer’s instructions for the ReverTra Ace qPCR RT Master Mix kit (Toyobo; Osaka, Japan). The cDNA product was utilized for qRT-PCR using the PowerUp™ SYBR™ Green Master Mix (Applied Biosystems, Waltham, MA, USA). Data analysis was performed using the Stratagene™ Mx3005P qPCR Instrument (Agilent, Santa Clara, CA, USA). qRT-PCR primers were designed using NCBI’s primer design tool (https://www.ncbi.nlm.nih.gov/tools/primer-blast/index.cgi?LINK_LOC=BlastHome, accessed on 10 October 2023) and are listed in Table 1. All primer synthesis was undertaken by Sangon Biotech (Shanghai, China), a biotechnology company.

### 4.5. Western Blotting

The cell lysates prepared in RIPA buffer were analyzed via immunoblotting, following a previously established method [57]. The antibody against GPX4 (catalog no. ab125066) was purchased from Abcam (Cambridge, UK). SLC7A11 (catalog no. 98051S) and GAPDH (catalog no. 5174S) were obtained from Cell Signaling Technology (Danvers, MA, USA). The uncropped original gel images of raw western blot data, with red boxes highlighting the selected results, are presented in Figure S1.

### 4.6. Iron Assay

We exposed 661W cells to 20 mM SI for 18 h. Then, the cells were treated with 5 μM FeRhoNox-1 (Goryo Chemical; Sapporo, Japan) and 2.5 μM Hoechst for 1 h at 37 °C. Following the treatment, the cells underwent triple washing with phosphate-buffered saline (PBS). Fluorescence imaging was conducted utilizing a Lecia confocal microscope (Leica; Wetzlar, Germany).

### 4.7. Lipid Peroxidation and ROS Levels Assay

Lipid peroxidation levels were assessed using the C11-BODIPY probe, and intracellular ROS production was measured with the H2DCFDA probe. We seeded 661W cells on glass slides and treated them with 20 mM SI for 18 h. After washing the cells three times with sterile PBS, they were incubated with 2.5 μM C11-BODIPY or 2.5 μM H2DCFDA and 2.5 μM Hoechst for 30 min (minutes) at 37 °C, followed by analysis using a confocal microscope (Leica; Wetzlar, Germany). Additionally, to further evaluate lipid peroxidation and intracellular ROS levels, 661W cells were pre-incubated with 2 mM NAC or 50 μM GSH for 2 h, followed by exposure to 20 mM SI for 18 h. The cells were then incubated separately with 2.5 μM C11-BODIPY or 2.5 μM H2DCFDA for 30 min at 37 °C, and lipid peroxidation and intracellular ROS levels were analyzed separately by flow cytometry (BD FACSMelody, BD Biosciences, Franklin Lakes, NJ, USA). Note that the C11-BODIPY probe is lipophilic and easily enters the cell membrane. Once oxidized, the probe maintains its lipophilicity and does not spontaneously leave the lipid bilayer. Upon lipid peroxidation, the polyunsaturated butadiene part of the probe is oxidized, and its maximal emission wavelength shifts from 590 nm to 510 nm, reflecting the level of lipid peroxidation in the membrane.

### 4.8. Data Analysis and Statistics

All results were analyzed using GraphPad Prism software (Version 8.0; La Jolla, CA, USA). All quantitative variables were expressed as the mean ± standard deviation (SD) of at least three independent experiments. Comparisons between two groups were performed using Student’s *t*-test, while comparisons among multiple groups were performed using one-way ANOVA followed by Tukey’s multiple comparison test. In all cases, a *p* value < 0.05 was considered statistically significant.

## Figures and Tables

**Table 1:** The amplified primer sequences.

Genes	Species	Forward Primer (5′→3′)	Reverse Primer (5′→3′)
*PTGS2*	mouse	AATGTATGAGCACAGGATTTGACC	TGTCAGCACATATTTCATGATTAAACTTCG [58]
*SLC7A11*	mouse	GGTCAGAAAGCCAGTTGTGG	AGTATGCCCTTGGGGGAGAT
*GAPDH*	mouse	AGGTCGGTGTGAACGGATTTG	TGTAGACCATGTAGTTGAGGTCA [58]

## Data Availability

The data presented in this study are available on request from the corresponding author.

## Supplementary Materials

The following supporting information can be downloaded at: https://www.mdpi.com/article/10.3390/ijms26052334/s1.
