# Supplementary material for: Sodium Iodate-Induced Ferroptosis in Photoreceptor-Derived 661W Cells Through the Depletion of GSH
Source: Int J Mol Sci. 2025 Mar 5;26(5):2334. doi: 10.3390/ijms26052334 (PMC11900459; doi:10.3390/ijms26052334)
Supplement: Supplementary file 1 [file ijms-26-02334-s001.zip › ijms-3405501-supplementary.pdf]

Supplementary Materials

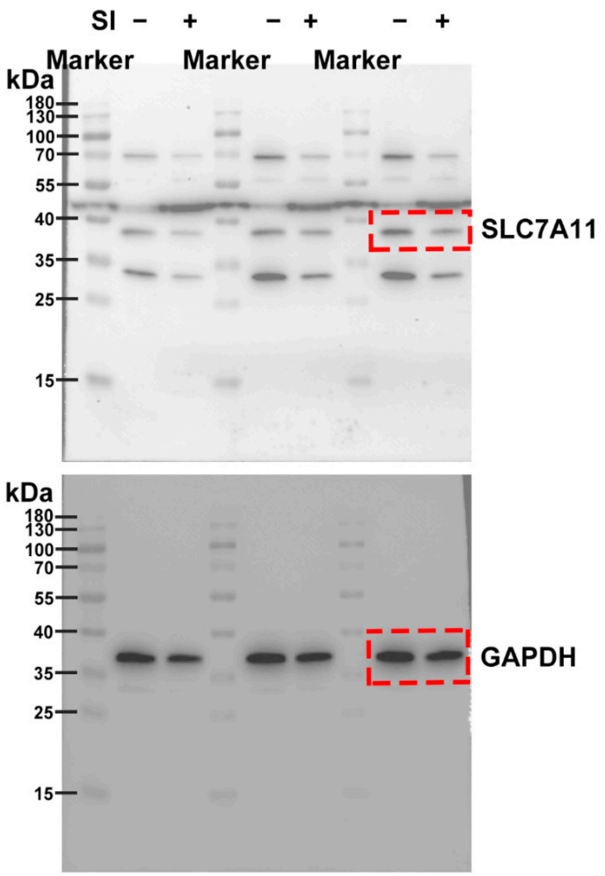

Figure 5B

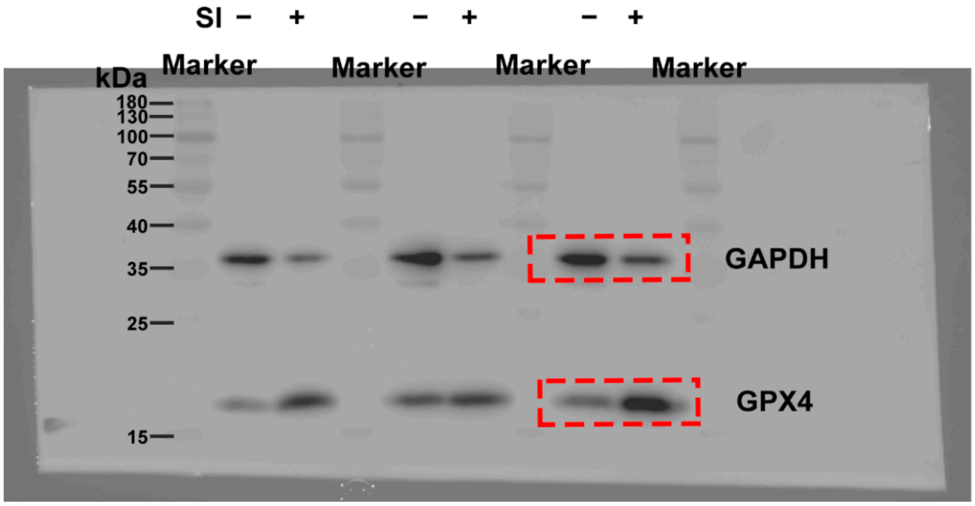

Figure 5E

Figure S1. Raw western blot data. Selected western blot results are indicated by the red boxes.
